# Modeling the Th17 and Tregs Paradigm: Implications for Cancer Immunotherapy

**DOI:** 10.3389/fcell.2021.675099

**Published:** 2021-05-07

**Authors:** Karla F. Corral-Jara, Gonçalo Rosas da Silva, Nora A. Fierro, Vassili Soumelis

**Affiliations:** ^1^Computational Systems Biology Team, Institut de Biologie de l’Ecole Normale Supérieure, CNRS UMR 8197, INSERM U1024, Ecole Normale Supérieure, PSL Research University, Paris, France; ^2^School of Biological Sciences, Queen’s University Belfast, Belfast, United Kingdom; ^3^Department of Immunology, Biomedical Research Institute, National Autonomous University of Mexico, Mexico City, Mexico; ^4^Université de Paris, INSERM U976, France and AP-HP, Hôpital Saint-Louis, Immunology-Histocompatibility Department, Paris, France

**Keywords:** Th17, Tregs, mathematical models, omics data, tumor microenvironment

## Abstract

CD4 + T cell differentiation is governed by gene regulatory and metabolic networks, with both networks being highly interconnected and able to adapt to external stimuli. Th17 and Tregs differentiation networks play a critical role in cancer, and their balance is affected by the tumor microenvironment (TME). Factors from the TME mediate recruitment and expansion of Th17 cells, but these cells can act with pro or anti-tumor immunity. Tregs cells are also involved in tumor development and progression by inhibiting antitumor immunity and promoting immunoevasion. Due to the complexity of the underlying molecular pathways, the modeling of biological systems has emerged as a promising solution for better understanding both CD4 + T cell differentiation and cancer cell behavior. In this review, we present a context-dependent vision of CD4 + T cell transcriptomic and metabolic network adaptability. We then discuss CD4 + T cell knowledge-based models to extract the regulatory elements of Th17 and Tregs differentiation in multiple CD4 + T cell levels. We highlight the importance of complementing these models with data from omics technologies such as transcriptomics and metabolomics, in order to better delineate existing Th17 and Tregs bifurcation mechanisms. We were able to recompilate promising regulatory components and mechanisms of Th17 and Tregs differentiation under normal conditions, which we then connected with biological evidence in the context of the TME to better understand CD4 + T cell behavior in cancer. From the integration of mechanistic models with omics data, the transcriptomic and metabolomic reprograming of Th17 and Tregs cells can be predicted in new models with potential clinical applications, with special relevance to cancer immunotherapy.

## Introduction

CD4 + T cell differentiation requires a combination of external signals to induce changes at the membrane, cytoplasmic and nuclear levels. This requires the activation of complex gene regulatory networks, involving transcriptional and non-coding RNA, cell signaling and epigenetic regulation (Christie and Zhu, 2014; Schmidl et al., 2018). This process of differentiation is also supported by distinct metabolic reaction networks (Buck et al., 2015).

This can be shown by how Th17 and Tregs CD4 + T cell phenotypes play a central role in cancer, and how to the conditions within the tumor microenvironment (TME) can lead to a deregulation and hijacking of these T cell networks for tumor proliferation (Guéry and Hugues, 2015). Flexibility between Th17 and Tregs cells is well-documented in the TME (Diller et al., 2016). For instance, Th17 cells have been shown to progressively transdifferentiate into IL-17A^+^FOXP3^+^ and IL-17A^–^FOXP3^+^ T cells during tumor development (Downs-Canner et al., 2017).

With the recent increase of immunotherapy as a promising therapeutic option against cancer, the regulation and differentiation of Th17 and Tregs cells has gained recognition as essential part of this equation (Zanetti, 2015; Sadozai et al., 2017). Elucidating the factors underlying changes in Th17 and Tregs networks will potentiate the development of innovative therapeutic strategies by manipulating metabolic and transcriptional intracellular pathways (Kim et al., 2018; Rivadeneira and Delgoffe, 2018).

The evolution of the field of systems biology has led to several advances in the mechanistic comprehension of the immune system (Smolke and Silver, 2011; Calder et al., 2018). Systems biology heavily relies on expansive, well-curated databases and prior literature, so the field has benefited from the integration of expanding omics technologies for several years (Karahalil, 2016). Metabolomics in particular, often via incorporation into existing models, are being increasingly integrated into both model development and new therapeutic/analytical approaches (Misra et al., 2017; Chong et al., 2018).

In this review, we address the mechanisms behind the decisions governing Th17 and Tregs cell fate, as explained by a systems biology point of view, through *in silico* simulations/predictions and their relation with the TME. We then offer an explanation of the biological adaptability of CD4 + T cells as the basis for understanding Th17 and Tregs plasticity. Subsequently, we present a recompilation of the molecular mechanisms behind Th17 and Tregs function, highlighted on theory-data-driven models, and connect this information with biological evidence in the context of the TME. Finally, we describe how cancer immunotherapeutic applications have been developed based on mathematical modeling strategies and omics data integration.

## Gene Regulatory Mediating CD4 + T Cell Differentiation

**FIGURE 1:**
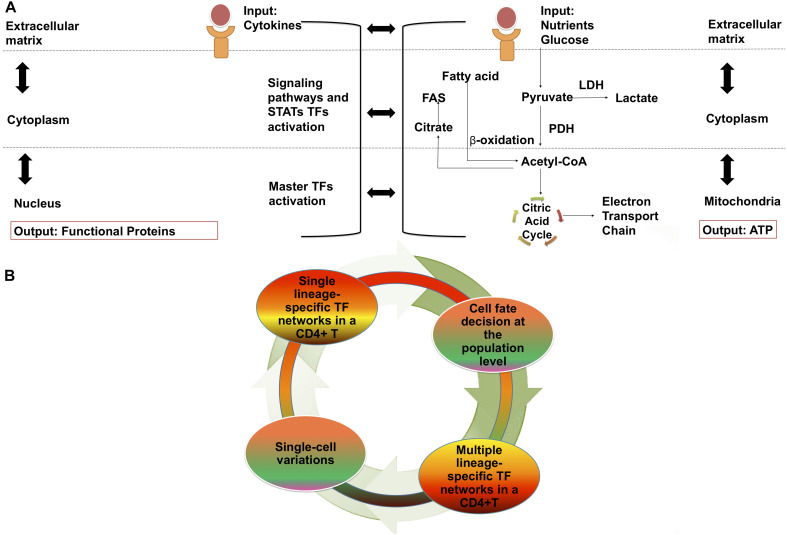
CD4 + T cell differentiation: Cellular regulation layouts and dynamism of transcriptional and metabolic networks. **(A)** Levels of cellular regulation in gene regulatory networks. Membrane: TCR, cytokines and binding of co-stimulatory molecules. Cytoplasm: signaling pathways and activation of STATs. Nuclear: activation of master TFs and transcription of functional proteins. And levels of cellular regulation in metabolic networks. Membrane: nutrients binding to receptors. Cytoplasm: activation of metabolic fluxes. Mitochondria: activation of the electron transport chain for the generation of ATP. **(B)** CD4 + T cell differentiation and network components dynamic. Under different external stimuli changes occurring on a intracellular level are observed. Among a set of cells that receives the same signal, individual cell variability is also presented. STATs, signal transducer and activator of transcription; FAS, fatty acid synthesis; LDH, lactate dehydrogenase; PDH, pyruvate dehydrogenase; ATP, adenosine triphosphate; TF, Transcription factor.

Th17 cells belong to the effector CD4 + T cell lineage (Teffs), while periphery-induced regulatory T (iTregs) and thymic regulatory T cells (tTregs) belong to the regulatory lineage. CD4 + T cells are regulated across three main regulatory layers; the cell membrane, cytoplasm, and nucleus (DuPage and Bluestone, 2016), with molecular and other signaling pathways occurring within and between these layers, and giving rise to gene regulatory networks (GRN) (Figure 1A), that contribute to the expression of genes involved in metabolic networks (Klosik et al., 2017; Hiemer et al., 2019) (Figure 1A).

**FIGURE 2:**
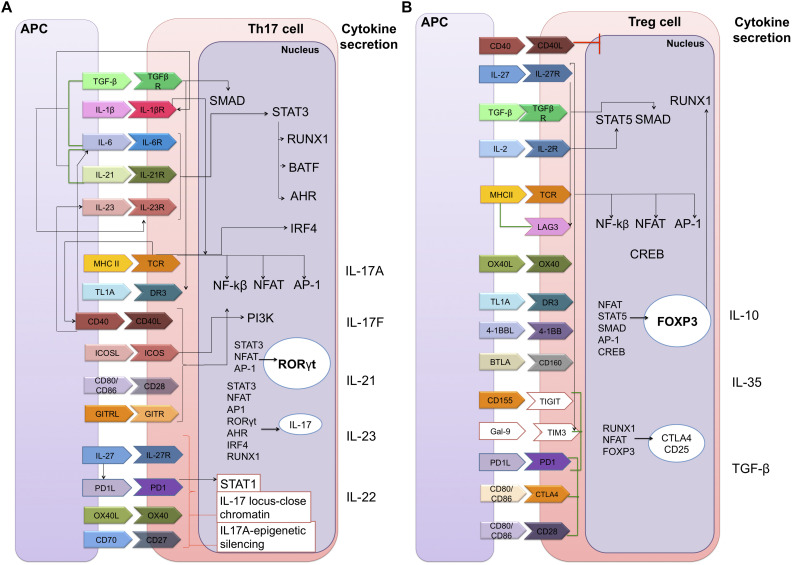
Static gene regulatory networks of CD4 + T cell differentiation: Th17 and Tregs. Each CD4 + T cell subset can be defined by their distinct abilities to sense, program and function in the control of specific pathogen or immune pathologies. The inductive cytokines, polarizing transcription factors and cytokines or chemokine receptors that are characteristic of each subset are shown. **(A)** Th17 cell differentiation. The Th17 subset is a key mediator of the inflammatory response; its functional attributes are the recruitment of neutrophils that are necessary for the clearance of extracellular bacterial and fungal infections. **(B)** Regulatory T cell differentiation. Tregs are vital to maintaining balance in the system since they allow tolerance and prevent autoimmune diseases by suppressing the function of Effector T cells. Membrane components: TGF-β, transforming growth factor beta; TGFβR, TGF-beta receptor; IL-1β, interleukin 1-beta; IL-1βR, interleukin 1-beta receptor; MHC II, major histocompatibility complex class II; TCR, T-cell receptor; TL1A, TNF-like ligand A1; DR3, death receptor 3; CD40, cluster of differentiation 40; CD40L, CD40 ligand; ICOS, inducible T-cell co-stimulator; GITR, glucocorticoid-induced TNFR family related gene; PD1, programed cell death protein 1; OX40, tumor necrosis factor receptor superfamily, member 4; LAG3, lymphocyte-activation gene 3; 4-1BB (or CD137). BTLA, B- and T-lymphocyte attenuator; TIGIT, T cell immunoreceptor with Ig and ITIM domains; Gal-9, galectin 9; TIM3, T-cell immunoglobulin and mucin-domain containing-3; CTLA4, cytotoxic T-lymphocyte-associated protein 4. Nuclear components: SMAD, smad family members; STAT3, signal transducer and activator of transcription 3; RUNX1, runt-related transcription factor 1; BATF, basic leucine zipper ATF-like transcription factor; AHR, aryl hydrocarbon receptor; IRF4, interferon regulatory factor 4; NF-kβ, nuclear factor-kappa B; NFAT, nuclear factor of activated T-cells; AP-1, activator protein 1; PI3K, phosphoinositide 3-kinases; RORγt, RAR-related orphan receptor gamma; CREB, C-AMP response element-binding protein; FOXP3, forkhead box P3. Extracellular secretion: IL-17A, IL-17F, IL-21, IL-23, IL-22, IL-10, IL-35, and TGF-β. Black lines means a positive direct interaction. Red lines are negative direct interactions and green lines indicate a bibliographic support on the synergistic function of the components in the differentiation of cellular subtypes.

The differentiation processes of these cells are also known, though not fully-established; CD4 + T cell signal differentiation is initiated in the T cell receptor (TCR) pathway, with cytokines and co-stimulatory molecules binding their receptors to the cell membrane. This then activates signaling pathways in the cytoplasm, which act as an intermediary between the membrane and the nucleus, in which the endpoints of these pathways will induce an overexpression of specific transcription factors (TFs). These TFs will then induce a particular CD4 + T cell subtype cytokines signature (Christie and Zhu, 2014) (Figure 2).

Previously, it was thought that the process of differentiating toward each individual CD4 + T cell subtype was modulated by a single lineage-specific TF and mutually inhibited by the others (Zheng and Flavell, 1997; Szabo et al., 2000). However, it has since been discovered that a CD4 + T cell lineage-specific TF could be expressed among multiple CD4 + T cell subtypes, introducing the concept of transcription factor co-expression (Evans and Jenner, 2013). Similarly, gene regulatory network variability at the single-cell level has been observed (Padovan-Merhar and Raj, 2013). A single CD4 + T cell may acquire different molecular phenotypes associated with transcriptomic and proteomic fluctuations (Vieira Braga et al., 2016). This adds significant complexity to our understanding of differentiation in these cells.

## CD4 + T Cell Differentiation and Metabolic Flux Network Adaptations

Similarly, CD4 + T cells react differently to different nutritional stimuli, activating different metabolic routes in the cytoplasm, such as glucose and its promotion of glycolysis. In most cases, in contrast to GRN, metabolic networks terminate in the mitochondria with the generation of adenosine triphosphate (ATP), the main energy source for the cell (Shyer et al., 2020).

There have been continuous efforts to define the metabolic hallmarks for each CD4 + T cell subtype. In general, CD4 + effector T cells prioritize glycolysis and lactate production under aerobic conditions. Conversely, Tregs immediately undergo fatty acid oxidation (FAO) leading the generation of ATP (Wang and Solt, 2016). Specifically, murine tTregs engage in glycolysis and glutaminolysis at levels comparable to effector T cells, despite expressing forkhead box 3 (FOXP3) (Priyadharshini et al., 2018), while murine iTregs have been shown to preferentially use FAO, despite the fact that human iTregs heavily rely on glycolysis (Pacella et al., 2018). Fatty acid synthesis (FAS) is an anabolic hallmark of CD4 + effector T cells, but not of Tregs, which require fatty acid uptake (FAU) (Buck et al., 2015; Howie et al., 2018).

These metabolic networks do not remain static during CD4 + T cell differentiation, and adapt in response to diverse stimuli. Instances of CD4 + effector T cells activating/relying on FAO and Tregs differentiating through glycolysis have both been documented. For example, CD4 + effector T cells rely on FAS and glutaminolysis as principal sources of energy in response to low glucose levels, but their normal functions and their capacity to synthesize IFN-γ are both compromised (Ecker et al., 2018). Simultaneously, Tregs take advantage of glucose scarcity and lactate abundance to undergo differentiation (Angelin et al., 2017). However, under certain conditions, Tregs rely on glycolysis to maintain their expression of FOXP3 and potentiate their suppressive functions (Fan and Turka, 2018). This evidence strongly suggests that, similar to GRN, metabolic networks are flexible and widely stimulus-dependent.

## Mutual Effects of Gene Regulatory Networks and Metabolic Networks in CD4 + T Cell Differentiation

The activation of the TCR signaling pathway in the cytoplasm acts as an intermediate domain between the GRN and metabolic networks (Klosik et al., 2017). This pathway is responsible for the activation of the phosphatidylinositol 3-kinase/mammalian target of rapamycin/protein kinase B (PI3K/mTOR/AKT) pathway, which is a key component of CD4 + T cell metabolism. This pathway involves the phosphorylation of phosphatidylinositol 4,5-bisphosphate (PIP2) by PI3K, converting it to phosphatidylinositol (3,4,5)- trisphosphate (PIP3), in a reaction negatively regulated by phosphatase and tensin homolog (PTEN) phosphatase. PIP3 promotes mTOR complex 2 (mTORC2) activation to phosphorylate AKT (Carlson et al., 2009; Liu et al., 2015).

Following TCR engagement, two mutually inhibited molecules, mTOR and AMP-activated protein kinase (AMPK) are expressed. mTOR is promoted in CD4 + effector T cells, while AMPK is expressed in Tregs. mTOR, a key signaling protein, is also related to the activation of metabolic processes such as glycolysis via Hypoxia Inducible Factor subunit alpha (HIF-1α) and FAS via sterol regulatory element-binding protein 2 (SREBP2), while AMPK can sense the cellular AMP/ATP ratio and respond to low energy conditions, inhibiting FAS and promoting FAO (Salmond, 2018).

Gene regulatory networks intermediates can also induce metabolism-related gene activation and vice-versa; metabolism-related genes regulate the transcriptional fingerprints on CD4 + T cells, since some metabolic pathway-related enzymes are transported to the nucleus and interact with mRNAs, thus impacting their stability and translation. For example, the glycolytic enzyme Gapdh in murine T cells can suppress the translation of IFN-γ by binding to the 3-UTR in mRNA (Chang et al., 2013).

Small variations in these gene and metabolic networks will result in a different immune response (Figure 1B). These significant differences in the mechanisms on which separate immune cell lines rely for energy production and the ways in which they react to external stimuli (especially from glucose and lactate) are also of special interest for cancer immunotherapy. Many cancers are known to be metabolically aberrant, causing significant alterations to their own biochemical composition and that of their TMEs (Ganapathy-Kanniappan, 2017). Competition for glucose (glycolysis) and mitochondrial metabolism between T cells and tumor cells have been identified as key events in determining the success of anti-tumor T cell activation (Yin et al., 2019), and the harting and “taming” of these metabolic traits have long been points of interest for cancer immunotherapy.

## Th17 and Tregs Balance Approached Through Systems Biology: Multiple Cell Regulatory Layers

The non-linear, cooperative and stochastic nature of the interactions in the immune system can make it difficult to delineate a mechanistic understanding of T cell differentiation, and also makes it more difficult to implement this knowledge into improving cancer immunotherapy outcomes. Computational modeling is increasingly involved in confronting these challenges, bridging the gaps left by unclear immunological processes (Chakraborty, 2017). An inspection of the published literature indicates that, within the field of systems biology applied specifically to understanding CD4 + T cells, a significant portion of studies looked at differentiation or immunotherapy implications.

Systems biology methodologies aim to predict how changes in the concentration of a particular component, or in the efficiency levels of a function relating to that component, will influence the overall activity of the system (Germain et al., 2011). Combining the organization-based approach of theory-driven models (to identify novel interactions) with the amount of data and the novelty of the network component obtained from data-driven models, highly predictive, hybrid models are ultimately expected to be constructed, with which we could achieve a better understanding of CD4 + T cells in TME context (Carbo et al., 2014; Morel et al., 2017). The goal is to find the appropriate balance between the oversimplification of system descriptions and inclusion of overly complex details (Klosik et al., 2017).

CD4 + T cell knowledge-based models are presented in the following sections, as means to identify Th17 and Tregs regulatory elements, including feedback loops and feed-forward loops in multiple cell layers, with the goal of finding their connections in a tumoral context.

## Modeling of Th17 Cells and Tregs at the Membrane Level for Understanding a TME

On the basis of model simulations, it has been concluded that both Tregs and Th17 cells are highly plastic and labile across different environmental conditions (Mendoza and Xenarios, 2006; Naldi et al., 2010; Mendoza, 2013). In addition, the behavior of master TFs has been modeled, leading to new discoveries such as FOXP3 expression being lost or transient in the absence of IL-2, while the expression of RORγt is more robust (Naldi et al., 2010; Hong et al., 2011). During differentiation, the Tregs phenotype passes through an additional intermediate state where they produce IL-2 before “activating” PTEN, enabling permanent activation of FOXP3 (Miskov-Zivanov et al., 2013).

However, Tregs are prevalent in nearly all cancers and act as immunosuppressive regulators of the body’s immune response making them a unique obstacle in cancer immunotherapy. The precise molecular mechanisms that guide Tregs cell stability in tumors remain elusive, but a cell-intrinsic role of the alarmin interleukin (IL)-33-ST2 axis in the functional stability of Tregs cells in the TME has been identified (Hatzioannou et al., 2020); more specifically, a feedback loop in which conventional mouse CD11c(+) dendritic cells (DC) stimulated by IL-33 secrete IL-2 to selectively expand IL-33R [ST2(+)]-suppressive CD4(+)Foxp3(+) Tregs was found (Matta et al., 2014). This suggests an important role for external cytokines in the mediation of CD4 + T cells in the TME.

Models of regulatory networks aimed at determining how external stimuli are processed to determine CD4 + T cell responses have also been developed (Naldi et al., 2010; Abou-Jaoudé et al., 2015). According to these simulations, several differentiated Th subtypes can be reprogramed from an initial state into various other subtypes by sequentially using proper environmental input conditions. Similarly, in response to varied cytokine mixtures, cells co-express lineage-specific proteins at diverse levels, such that the cell population spans a continuum of intermediate states between canonical cell phenotypes (Eizenberg-Magar et al., 2017).

In Th17 models, pitchfork bifurcations with TGF-β concentrations have been analysed by several teams. Increasing the TGF-β concentration caused the transformation of FOXP3 single-positive Tregs into RORγt-expressing Th17 cells and doubly positive cells expressing FOXP3high RORγthigh (Hong et al., 2011; Martinez-Sanchez et al., 2018). TGF-β can have a determining impact on T cells within the TME since it could promote Tregs or Th17 expansion and present a pleiotropic function (Dahmani and Delisle, 2018).

In the TME, Th17-Tregs differentiation is typically directed by multiple external signals driving opposing regulatory pathways. Studies on the effects of combining lineage-specifying cytokines on polarization and the acquired non-canon cell phenotypes are becoming more common. A data-driven computational model predicted a new function for IL-12 as an inducer of IL-17F, but not of IL-17A in an IL-1β signaling context (Grandclaudon et al., 2019). Subsequently, a hierarchical additive regression model was constructed, providing a framework for the prediction of cellular responses to new cytokine combinations and doses. In this case, the input conditions of TGF-β + IL-6 + IL-12 + IL-4 + IL-2 supported the *in silico* expression of IL-17A (Eizenberg-Magar et al., 2017).

IL-12 in particular has emerged as one of the most potent cytokines in mediating antitumor activity in a variety of preclinical models. IL-12 establishes a link between innate and adaptive immunity that involves different immune effector cells and cytokines depending on the type of tumor or the affected tissue (Tugues et al., 2015). Therefore, these mechanistic models could provide the first clues about the anti-tumoral response of IL-12, possibly through the induction of effector cytokines, such as IL-17A (Corral-Jara et al., 2021).

**FIGURE 3:**
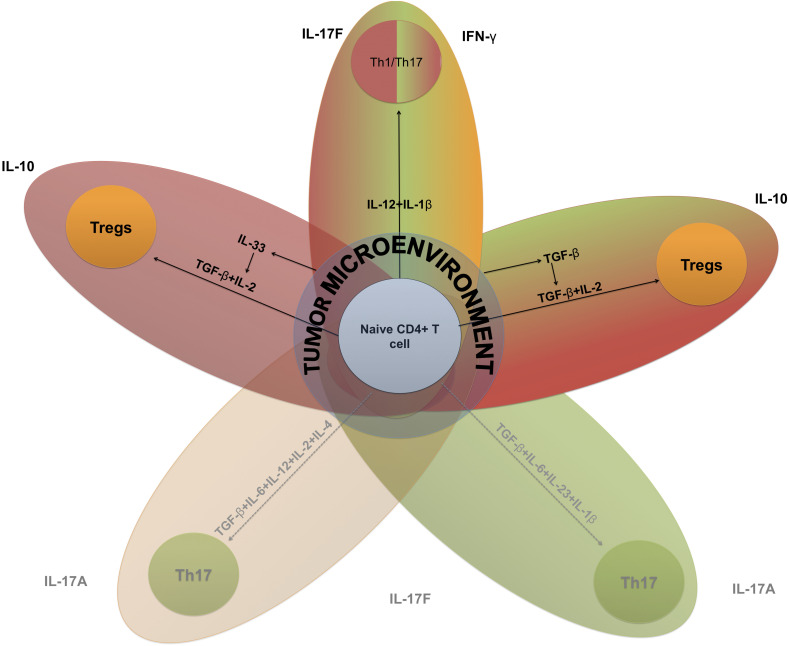
Key elements of Th17-Tregs differentiation based on systems biology-extracted information. Cytokine-driven T cell plasticity in a tumor microenvironment. The key inductive cytokines IL-12, IL-6, IL-4, IL-1β, I-L23, TGF-β, and IL-2, alone or in concert, can polarize naive CD4 + T cells toward different functions. This figure is intended to reveal the interconnectedness of these different programs based on the capacity of these inductive cytokines to promote polarization between subsets. These subsets include Th1–Th17, hybrid cells that produce IFN-γ and IL17 cytokines from a mixture of inductive cytokines characteristic of Th1 and Th17. TME, tumor microenvironment. Black lines represent positive interactions and dotted lines represent inhibited processes.

The Th17-Tregs balance has strong implications in autoimmunity and cancer immunotherapy (Knochelmann et al., 2018), and it may exhibit a range of sensitivities to altered concentrations of cytokines as they switch from one phenotype to another (Figure 3).

## Modeling of Th17 Cells and Tregs at the Transcriptional Level for Understanding a TME

A model aimed at understanding the mechanisms mediated by TFs in Th17 cells and Tregs has corroborated that increasing the concentrations of PPARγ in Th17 cells would lead to the downregulation of RORγt and IL-17 and the upregulation of FOXP3 upon the addition of external TGF-β and external IL-6 *in silico* (Carbo et al., 2013).

PPARγ induces the expression of genes associated with FAU, a feature of Tregs, and controls glucose oxidation by inhibiting pyruvate dehydrogenase (PDH) (Howie et al., 2018). The inhibition of PDH by pyruvate dehydrogenase kinase 1 (PDHK1) is required for Th17 cell development (Gerriets et al., 2015). In contrast, Th1 and Th2 cells are inhibited by TOFA, an inhibitor of ACC1 (a master enzyme of FAS, a feature of CD4 + effector T cells); however, there are discrepancies regarding the Th17-TOFA effect (Berod et al., 2014; Angela et al., 2016), indicating that PPARγ and FAU may also support a Th17 cytokine signature (Nicholas et al., 2017) and be a potential mechanism to consider in immunotherapeutic approaches.

Lipids are a key part of the TME (Luo et al., 2018), and fatty acid binding proteins (FABPs) play important roles in fatty acid uptake from the microenvironment. FABP5 is one of the most highly expressed FABPs in T cells (Rolph et al., 2006), and its inhibition has been shown to minimize IL-17 cytokine production and skew T cells toward a Tregs phenotype *in vitro* (Field et al., 2020). FABP5 has also been associated with the development of diverse types of cancer (Carbonetti et al., 2019), indicating the importance of considering metabolic aspects of CD4 + T cells in the TME.

## Modeling of Th17 Cells and Tregs at the Signaling Level for Understanding a TME

Signaling and metabolic modulations of Th17-Tregs differentiation are directly related to the intensity of TCR signaling. Computational modeling assays have been performed to determine how the TCR signal strength engages alternate signaling networks to control cell fate decisions. Weak TCR signals generated elevated PIP2 and reduced PIP3 levels, and high PIP3 levels activate mTOR/AKT signaling completely toward glycolisis (Hawse and Cattley, 2019). Using a model of multiple interleukin 2 tyrosine kinase (ITK) signaling circuits, a tyrosine kinase required for full TCR-induced activation of mTOR, it was predicted that inositol (1,3,4,5) tetrakisphosphate (IP4) might promote ITK binding to PIP3 through cooperative allosteric selection (Mukherjee et al., 2013).

Analyzing Th17 and Tregs differentiation, it has been demonstrated that CD4 + T cells deficient in ITK exhibit decreased IL-17A expression, decreased IL-2-induced phosphorylation of mTOR and increased FOXP3 induction due to PTEN activation (Gomez-Rodriguez et al., 2009, 2014). In the TME, data from a murine colon adenocarcinoma model demonstrated a decline in functionality of Tregs cells from *Itk*^–/^*^–^* mice, which further supports the connection between decreased ITK expression and decreased Tregs cell functionality (Lutsiak et al., 2008).

Revu et al. (2018), stated that CD28 co-stimulation suppresses the induction of the Th17 cell transcriptional program, activating the mTOR/AKT pathway to high levels, indicating that some mTOR/AKT activation is required despite high mTOR/AKT activity suppressing Th17 cell development (Revu et al., 2018). This supposition suggests that the plasticity of Th17 and Tregs can be modulated by dedicated doses of external factors that have an effect on cell signaling pathways.

**FIGURE 4:**
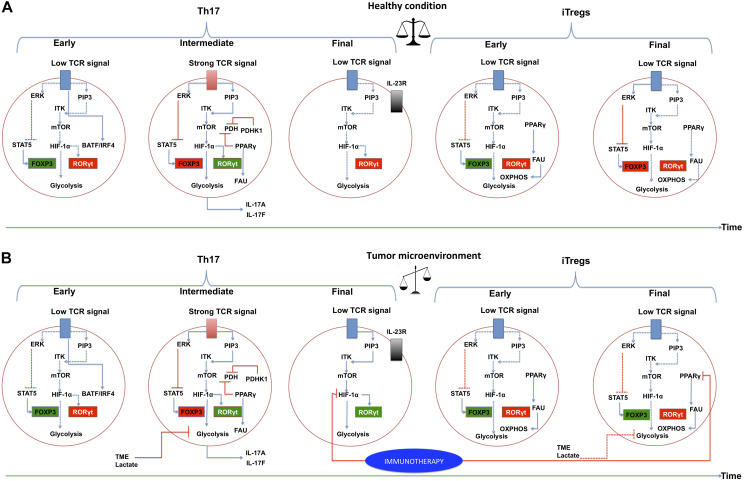
The PI3K–AKT–mTOR pathway and metabolic programs converge to regulate the differentiation of inflammatory versus regulatory T cells in a tumor microenvironment. Links between extracellular cues, T-cell receptor (TCR) strength, the phosphatidylinositol 3-kinase (PI3K)–AKT–mammalian target of rapamycin (mTOR) pathway, metabolic programs and gene regulation are depicted. **(A)** Normal conditions. Three stages of differentiation over time are shown for Th17, at the end of the timeline, Tregs differentiation is presented to indicate that these require more time to differentiate. IL-2-inducible T-cell kinase (ITK) and TCR signaling play a critical role in regulating the expression of transcription factor hypoxia-inducible factor (HIF-1α) by functioning as a rheostat that determines the extent of activation of PI3K and mTOR-activated pathways. HIF-1α is activated in intermediate and deactivated in final steps of Th17 differentiation. A balance between Th17 cells and Tregs is achieved and they can co-exist in the time. PPARγ in Tregs should be deactivated in a specific time. **(B)** Tumor microenvironment conditions. We suggest a deregulation of the signaling pathways in Th17 cells and Tregs. It is likely that HIF-1α is not inactivated in a tumor microenvironment, leading to a higher expression of RORγt, and generating an exhausted Th17. Likewise, PPARγ may not be deactivated in Tregs, leading to functional Tregs. Immunotherapy proposals could be directed at targets such as HIF-1α and PPARγ. Activated interactions between components are indicated by solid lines, whereas inactivated links between proteins are denoted with dashed lines. The blue lines indicate positive direct interactions between the components. Red lines indicate direct negative interactions. The green squares indicate that the component is active, the red square indicates that the component is inactive. ERK, extracellular-signal-regulated kinase pathway; PIP3, phosphatidylinositol (3,4,5)-trisphosphate; STAT5, signal transducer and activator of transcription 5; FOXP3, forkhead box P3; RORγt, RAR-related orphan receptor gamma; PDH, pyruvate dehydrogenase; PDHK1, pyruvate dehydrogenase kinase 1; PPARγ, peroxisome proliferator-activated receptor gamma; FAU, fatty acid uptake; TME, tumor microenvironment.

Thymic regulatory T cells require a strong TCR signal since their metabolism is based on glycolysis, and iTregs require a low TCR signal due to their relying primarily on fatty acid metabolism. In the case of Th17 cells, there are discrepancies about the strength of the TCR signaling required to drive their differentiation (DuPage and Bluestone, 2016), as has been previously discussed. It is possible that, at the beginning of Th17 cell differentiation *in vivo*, a low TCR signal is required, leading to increases in STAT5 and PIP2, and a decrease in PIP3-ITK interactions. This will lead to a decrease in the activity of the mTOR pathway. At an intermediate time, a strong signal from the TCR is required, reversing the aforementioned processes. Upon the conclusion of this process, the TCR signal may decrease again (Figure 4A).

The TME has an intricate and profound effect on CD4 + T cell signaling pathways. For instance, lactate released by tumor and stromal cells generates extracellular acidity, and inhibits the PI3K/Akt/mTOR pathway, thus also inhibiting T cell glycolysis. Additionally, acidification of the TME impairs Teffs to a much greater extent compared to Tregs, mostly because Teffs acquire energy mainly through glycolysis, while Tregs can rely on FAO (Yin et al., 2019) (Figure 4B).

## Th17-Tregs Balance and Omics Data: a Complement to the Theoretical Model Approach

The intricately-linked networks by which CD4 + T cells differentiate have been broadly approached to date (Koch and Radtke, 2011), with a body of research being steadily built over several years and benefiting/accelerating largely based on factors such as the increased accessibility of DNA and RNA sequencing technologies (Frio, 2015).

Most of the current systems biology-based approaches derived from real biological/medical data are based on the genome, transcriptome or proteome of an organism, a situation partially attributable to these datasets being measured by established and tested methods, and the continued capacity of these methods to iterate and integrate new protocols, such as single-cell transcriptomics (Stubbington et al., 2017). Analyses of these datasets are slowly becoming more accurate and manageable due to the frequent release and update of new dedicated tools (Chong et al., 2018; Marco-Ramell et al., 2018) and, in addition to the increased resolution, throughput capacity and accuracy that might be expected over time, developments that enable the exploration of new paradigms, such as single-cell metabolomics (Zenobi, 2013).

## Th17 and Tregs Differentiation-Transcriptomics-Based Approach and TME Implications

Some teams have experimented with high-throughput data to construct a Th17 cell differentiation network, and reported that there are three transcriptional phases that control mouse Th17 cell differentiation: early, intermediate and late (Ciofani et al., 2012; Yosef et al., 2013). As proposed, there are also three possible metabolic phases of Th17 cell differentiation in which TCR-AKT-mTOR pathway activity is modulated. Determining the mechanisms of GRN-metabolic network integration is a promising concept in TME comprehension.

The early transcription phase is characterized by *cis* regions bound by BATF/IRF4 complex under TCR stimulation conditions (Th0 cells), and with promoted chromatin accessibility, binding strongly with STAT3, RORγt, c-Maf, and p300 in cells (Ciofani et al., 2012).

The transition to the intermediate phase is marked by the induction of RORγt and another TFs, known (*e.g.*, Ahr) and novel (*e.g.*, Trps1) (Yosef et al., 2013). RORγt is sensitive to changing environmental signals and is essential for licensing the expression of a select few loci; it functions as a rheostat to tune mRNA levels to those of a Th17-specific program. RORγt can also limit target expression, including that of the regulators of metabolism and quiescence, e.g., IL-10, HIF-1α, Egln3, Foxo1, and IL-7R (Ciofani et al., 2012).

HIF-1α is induced by mTOR and mediates glycolytic activity, thereby contributing to the Th17 cell or Tregs lineage switch (Shi et al., 2011). We suggest that, in the late stages of Th17 cell differentiation, the inhibition of the mTOR pathway and HIF-1α, may subsequently be required in order to ensure the transition toward the final stage of the process. During the conclusion of the differentiation step, Th17 cells induce IL-23R, which plays an important role in the late phase (Yosef et al., 2013).

It has been suggested that, during the late stage of Th17 differentiation, HIF-1α must be decreased. HIF-1α is highly expressed in Th17 cells, priming at physiological oxygen tension in the presence of inflammatory cytokines. HIF-1α plays a prominent role in Th17 cell differentiation and it helps recruit CBP/p300 to the RORγt transcription complex, but does not directly bind to the IL-17 promoter. Additionally, HIF-1α increases glycolysis by inducing the expression of glycolytic enzymes, which further contributes to Th17 development. However, HIF-1α also promotes carcinogenesis and is a prominent cancer target, and various HIF-1α inhibitors have been identified and are currently being studied for their efficacy in cancer therapy (Chou et al., 2016). Although Th17 cells are prevalent within the TME, their functional role in tumor immunity is controversial (Ye et al., 2013), and the three suggested steps of Th17 differentiation and HIF-1α participation could provide partial explanations.

## Th17 and Tregs Differentiation-Metabolomics-Based Approach and TME Implications

CD4 + T cell differentiation networks have been classified at the genomic and proteomic level (Zhu and Paul, 2010; Liston and Schlenner, 2015), but incorporating metabolism has proven difficult, since gene expression is not directly correlated to the activity of the encoded metabolic enzyme (Wang et al., 2017).

Metabolomics concerns the study of metabolites, the downstream results of the many processes in tissues and fluids that comprise the functions of life, and it is a relatively recent branch of the omics family of technologies. Despite the high cost and difficulty of metabolomic data interpretation, its possible integration into systems biology methodologies and its capacity to define CD4 + T cell phenotypic traits, as opposed to purely genotypic traits, make it an interesting tool (Misra et al., 2017; Trivedi et al., 2017).

CD4 + T cell differentiation is also known to be heavily influenced by external factors, such as the modulation induced by obesity, specific nutritional choices (Maruyama et al., 2011; Endo et al., 2015) and the presence and effects of the gut microbiota under physiological conditions (Frankel et al., 2017), giving increased importance to metabolomics as a valuable cellular phenotype-centered method for screening pathways and networks of exposotypes. Ideally, this will result in a more comprehensive systems biology approach (Rattray et al., 2018).

Metabolomics has been used to characterize the actions of Tregs. Specifically, short-chain fatty acids (SCFAs) produced by commensal microorganisms during starch fermentation have an immunomodulatory effect on the balance of pro- and anti-inflammatory cells. For instance, butyrate, a known histone deacetylase inhibitor, facilitates extrathymic generation of Tregs due to increased Foxp3 protein acetylation (Arpaia et al., 2013). Another SCFA, pentanoate, also shows potent histone deacetylase-inhibitory activity in CD4 + T cells and reduces IL-17A production and Th17 cell differentiation; however, it does not have an impact on Tregs development, despite being able to induce the production of IL-10 (a Tregs-promoting cytokine) in lymphocytes by reprograming their metabolic activity toward elevated glucose oxidation, supplying additional pentanoate-originated acetyl-CoA for histone acetyltransferases, and allowing IL-10 transcription after pentanoate-triggered enhancement of mTOR activity (Luu et al., 2019). High blood butyrate and propionate levels are also associated with resistance to CTLA-4 blockade and higher proportion of Tregs cells in TME (Coutzac et al., 2020).

Using metabolomics data, a key bifurcation point between T cell glycolytic and oxidative metabolism was discovered, specifically, the TCR-signaling-dependent activation of PDHK1, an inhibitor of PDH, was found to be involved (Menk et al., 2018). PDHK1 plays a role in CD4 + T cell fate through mechanisms depending on the CD4 + effector T cell subtype, as indicated by the enzyme being expressed in Th17 cells but not in Th1 cells and being required for Th17 cell but not Tregs function *in vitro* (Gerriets et al., 2015). In addition, a differential coupling of serine metabolism based on lineage choices and the production of cytokines IL-17 or IFN-γ were demonstrated by a data-driven metabolic network in an untargeted metabolomics analysis, while targeting the serine pathway promoted Tregs lineage development (Andrejeva, 2018).

Differential T cell metabolism can be used as a target to produce a specific CD4 + T cell phenotype, due to its strong relation with GRN components. In addition to these metabolomics and systems biology studies focusing on physiology and function, these technologies have also been employed in the search for diagnostics and markers of disease status and outcome, giving them another layer of potential use within the field of cancer immunotherapy (Li H. et al., 2019).

## Immunotherapy Applications: Omics Data and Mathematical Models and Limitations

We have emphasized strong functional indicators of the differentiation and roles that Tregs and Th17 cells have in the progression and in the treatment of cancer, as well as in how they react to external stimuli. The Th plasticity between these also plays an important role in the TME. This plasticity/reprograming can occur simultaneously or sequentially in response to specific microenvironmental cues to ultimately fuel complex immune interactions that participate in tumor progression (Jäger et al., 2009). This complex interplay adds new dimensions to the immunometabolism of T cells, and can present a hurdle for data interpretation.

The differentiated Tregs cells can be converted into Th17 cells under the influence of strong inflammatory conditions (Xu et al., 2007). For instance, hypoxia induced the expression of IL-17 in FOXP3^+^ Tregs in colorectal cáncer (CRC), FOXP3^+^IL-17^+^ T cells were then capable of inducing CRC-associated cell markers in bone marrow-derived mononuclear cells and drove the cells to be cancer-initiating cells (Yang et al., 2011).

It is also important to note that, while much of the literature on effector T cells in the TME focuses at least in part on Th17 and Tregs, due to how their balance is known to influence prognosis, the TME is populated by other T cell subsets with important roles. Th9 and IL-9 are similarly considered complementary to Th17 cells, and have similarly been found to promote cancer metastasis, potentially in combination with Th17. Accordingly, inhibition of IL-9 or IL-17 cytokines by neutralizing antibodies decreased epithelial-mesenchymal transition (EMT) and slowed lung cancer progression and metastasis (Salazar et al., 2020).

An anti-tumor property of Th1/Th17 hybrid cells have also been reported. The heightened effector function and prolonged persistence of Th1 and Th17 cells, respectively, are the key features of these hybrid cells. The enhanced anti-tumor cells were dependent on the increased NAD(+)-dependent activity of the histone deacetylase Sirt1 (Chatterjee et al., 2018). Th1, in similarly to Th17, depends on glutaminase activity and glutamine metabolism (Johnson et al., 2018). Glutamine metabolism affected histone modifications in human breast cancer cell lines, and the treatment of non-invasive epitelial and invasive mesenchymal breast cancer cell lines with a glutaminase inhibitor induce a downregulation of epigenetic regulatory genes, such as Sirt1. The interplay of metabolism and epigenetic regulatory mechanisms has become a focal point for a better understanding of cancer development and progression (Simpson et al., 2012; Renaude et al., 2020).

Similarly, Tregs have attracted attention due to their well-defined metabolic hallmarks, such as their reliance on FAO and oxidative phosphorylation for energy production and the centrality of mTORC1 for their maintenance. These features, have led to them also being targeted for metabolic modulation within the TME for therapeutic purposes, through targets such as TLR8. TLR8 signaling-mediated reprograming of glucose metabolism and function in human Tregs cells can enhance anti-tumor immunity *in vivo* in a melanoma adoptive transfer T cell therapy model (Li L. et al., 2019).

Singling out immunotherapy, for which one of the major modern challenges has been the balancing of patient benefit with a low toxicity profile, the fact that metabolites are the products and byproducts of cellular biochemical activities places them downstream of all other “omics” technologies; this makes them excellent biomarkers for disease and toxic stimuli/conditions within the body (Metallo and Vander Heiden, 2013; Zhang et al., 2015).

Factors such as the inherent variability of cancer and the complexity of the TME make it very difficult to detect a viable and reproducible metabolic fingerprint, especially when considering how certain components of the TME, such as Th17 cells and other subsets with IL-17 and IL-23, can help both fight (Muranski et al., 2008) and promote cancer growth (Numasaki et al., 2005; Dawod et al., 2020), but it is also notable that positive results have been achieved recently during attempts to find biomarkers of chemotherapeutic responses (Cardoso et al., 2018; Amin et al., 2019; Ghini et al., 2020). Biomarkers for response to immune checkpoint inhibitor therapy (ICI), such as the ratio of serum kynurenine/tryptophan, have shown promise in detecting negative outcomes for treatment in advanced melanoma and renal cell carcinoma patients treated with nivolumab, an antibody against programed cell death protein 1 (PD1) (Li H. et al., 2019). Metabolites are also routinely integrated (together with proteomics and transcriptomics) in proposed diagnostic tools for multiple types of cancer (Argelaguet et al., 2018; MacMullan et al., 2019).

The ubiquity of metabolomics data when studying immunotherapy is especially notable due to the presence of this technology in many of the most recent trends in the field, such as the acknowledgment of the importance of the microbiome (Gopalakrishnan et al., 2018), and how selectively-enriched fauna can influence treatment outcomes (Botticelli et al., 2020). Keen et al. (2018) also proposed the inclusion of high-throughput proteomics and metabolomics (in addition to genomic sequencing) in future large-scale studies that aim to show that ICI success is dependent on the microbiome to better characterize the mechanisms at play. The use of immunometabolomics is slowly uncovering new therapies and helping to better understand the behavior of these cells.

However, it is equally important to acknowledge that these options do not exist in isolation and serve best when incorporated with existing modalities. As an example of this, a recent single cells *in silico* based on single-cell RNA-Seq and flux balance analysis by Wagner et al. (2020) showed that, Th17 cell metabolic diversity reflects a balance between glycolysis and FAO, which is associated with pathogenicity. Pathogenic Th17 cells maintain higher aerobic glycolysis and TCA activity, whereas non-pathogenic Th17 cells oxidize fatty acids to produce ATP (Wagner et al., 2020). It agrees with our hypotheses proposed in previous sections, indicating that the processes of differentiation of Th17 and Tregs is changing over time and can go through various processes of activation or inhibition of signaling pathways (Figure 4).

This also reinforce the notion that, as useful as a metabolomics approach can be, it benefits from being incorporated into a larger toolkit that helps to make these results more context-sensitive. Mathematical modeling of cancer metabolism has been undertaken for decades (Markert and Vazquez, 2015) and has recently been an active part of drug discovery and therapeutic design (Sun et al., 2016; Roy and Finley, 2017), but the extreme complexity of the TME and the associated signaling and cross talk between lymphocytes and other cells has made precise modeling and interpretation very difficult, however, this situation is slowly improving due to systems biology integration (Pinu et al., 2019). For instance, a set of mathematical equations has been used to describe the pharmacodynamics of radiotherapy in combination with two immunotherapies, the blockers PD1-PDL1 axis and of the CTLA-4 pathway. This model explained several experimental results reported in preclinical and clinical settings, and paves the way for the efficient *in silico* design and optimization of combined anticancer therapies (Serre et al., 2016).

Despite some limitations in System Biology and omics approaches, including difficulties in collecting and processing data, lack of a suitably optimized immunometabolism-dedicated metabolite database, and potential technological limitations, there have been several promising advances in the disparate omics fields and continuous efforts are being taken to achieve their seamless integration on fundamental levels (Mitchell et al., 2015). This could potentially be beneficial for the study and treatment to other immune-mediated diseases, ranging from rheumatoid arthritis to multiple sclerosis and Alzheimer’s disease (Ferretti et al., 2016; Ciccocioppo et al., 2019), assuming the datasets generated are of high enough quality.

For example, simultaneous single-cell multiomics data collection will likely result in higher quality and more reproducible or directly comparable data sets. When coupled with efforts for further methodological and instrumental standardization, these data collection methods present unique opportunities to gain understanding on the fundamentals of CD4 + T cell differentiation and CD4 + T cell-focused applications in future immunotherapies.

## Discussion

The effect of Th lymphocytes on carcinogenesis may largely depend on the context of the tumor type and cancer stage, cytokine availability, receptor distribution, and crosstalk among different stimuli, cell types, and signaling pathways.

The complexity of CD4 + T cell function in TME has contributed to some of the limitations found in system biology interpretation, therefore, the complementarity of these methods with omics data will be necessary to obtain more precise hypotheses.

Intracellular network modeling has been used because of the importance of understanding that a range of dynamic cell population behaviors, including cellular synchronization, delays, and bimodal responses, can emerge from simple networks (Eizenberg-Magar et al., 2017; Thurley et al., 2018). The function of Th17 and Tregs modeling will allow for the prediction of the effect of these subpopulations on tumor development.

Omics integration is reaching its maximum point in the definition toward a better response and understanding of the TME. The integration of metabolomics has presented useful future applications to understand CD4 + T cell behavior in TME. In particular, it has recently produced promising results in the subcellular imaging of specific metabolite distributions (Pareek et al., 2020), revealing novel potential techniques for monitoring immune cell metabolic signals. However, few current examples of a full system biology integration with other omics data are known, despite promising clinical applications and toward a better CD4 + T cell function delineation in the TME (Lazarus et al., 2019; Kirshtein et al., 2020; Paterson et al., 2020).

## Author Contributions

KC-J and GR contributed equally to the writing of the manuscript. NF and VS contributed to its revision. All authors contributed to the article and approved the submitted version.

## Conflict of Interest

The authors declare that the research was conducted in the absence of any commercial or financial relationships that could be construed as a potential conflict of interest.
